# Case report: right radical nephrectomy with thrombectomy in a patient with renal tumor, retrohepatic inferior vena cava agenesis, and tumor extension to the azygos vein

**DOI:** 10.1093/jscr/rjaf351

**Published:** 2025-05-31

**Authors:** Mateo B Nolfi, Agustin Vaccaro, Melisa Amondarain, Patricio García-Marchiñena, Juan Pekolj

**Affiliations:** Department of General Surgery and Urology, Hospital Italiano de Buenos Aires Tte Gral. Juan Domingo Peron 4190, Buenos Aires, CP 1200, Argentina; Department of General Surgery and Urology, Hospital Italiano de Buenos Aires Tte Gral. Juan Domingo Peron 4190, Buenos Aires, CP 1200, Argentina; Department of General Surgery and Urology, Hospital Italiano de Buenos Aires Tte Gral. Juan Domingo Peron 4190, Buenos Aires, CP 1200, Argentina; Department of General Surgery and Urology, Hospital Italiano de Buenos Aires Tte Gral. Juan Domingo Peron 4190, Buenos Aires, CP 1200, Argentina; Department of General Surgery and Urology, Hospital Italiano de Buenos Aires Tte Gral. Juan Domingo Peron 4190, Buenos Aires, CP 1200, Argentina

**Keywords:** renal cell carcinoma, thrombosis, retrohepatic vena cava

## Abstract

Renal cell carcinoma accounts for 90% of malignant kidney tumors, but only 6% of patients typically present with associated thrombosis. We present the case of a male patient with a vascular anoma ly characterized by agenesis of the retrohepatic portion of the inferior vena cava. The hepatic veins drain directly into the right atrium, while the azygos vein serves as an alternative venous pathway, draining into the superior vena cava. A thrombus extends through the azygos vein into its intrathoracic portion. This vascular variant is associated with a right renal tumor and thrombosis involving the azygos vein. The aim is to highlight the importance of multidisciplinary management, neoadjuvant therapy, and surgical treatment to reduce morbidity and mortality in these cases.

## Introduction

Renal tumors account for 2% of all solid tumors, being the sixth most common cancer in men and the eighth in women. Renal cell carcinoma (RCC) represents 90% of malignant kidney tumors and 3% of all cancer-related deaths worldwide, with 30% of diagnoses at locally advanced or metastatic stages of disease. Neoplastic extension of the primary tumor into vascular structures leads to the formation of a tumor thrombus (TT), which extends into the main renal vein and may progress into the inferior vena cava (IVC). This occurs in ~6%–10% of patients with RCC. Among these cases, renal vein thrombosis is observed in up to 44%, and in 1%–4% the thrombus may reach the right atrium [1, 2].

Neoadjuvant therapy was initially added to the standard treatment regimen for RCC with the aim of reducing tumor burden prior to surgical resection, simplifying the surgical approach in more complex cases, and has shown a decrease in thrombus height in 42%–52% of patients in some series [3].

**Figure 1:**
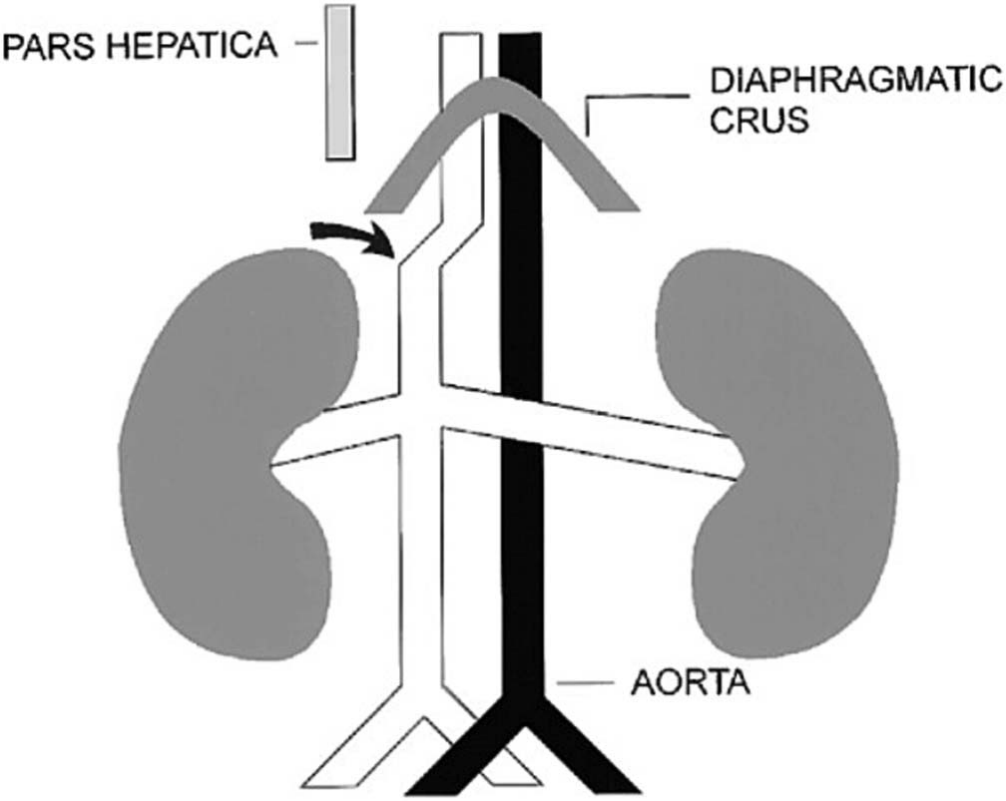
Schematic shows lack of contiguity between the prerenal segment of the IVC (arrow) and the hepatic segment. The vessel parallel to the aorta under the crus is the azygos vein [11].

On the other hand, with regard to vascular malformations of the IVC, agenesis of the IVC with continuation into the azygos vein (see Fig. 1) is a rare congenital malformation that affects 0.6% of the population [4, 5].

The main goal of this case is to describe the management of RCC with associated thrombosis, demonstrating the benefit of neoadjuvant therapy in a particular case of RCC with retrohepatic IVC agenesis and azygos vein thrombosis.

## Case report

**Figure 2:**
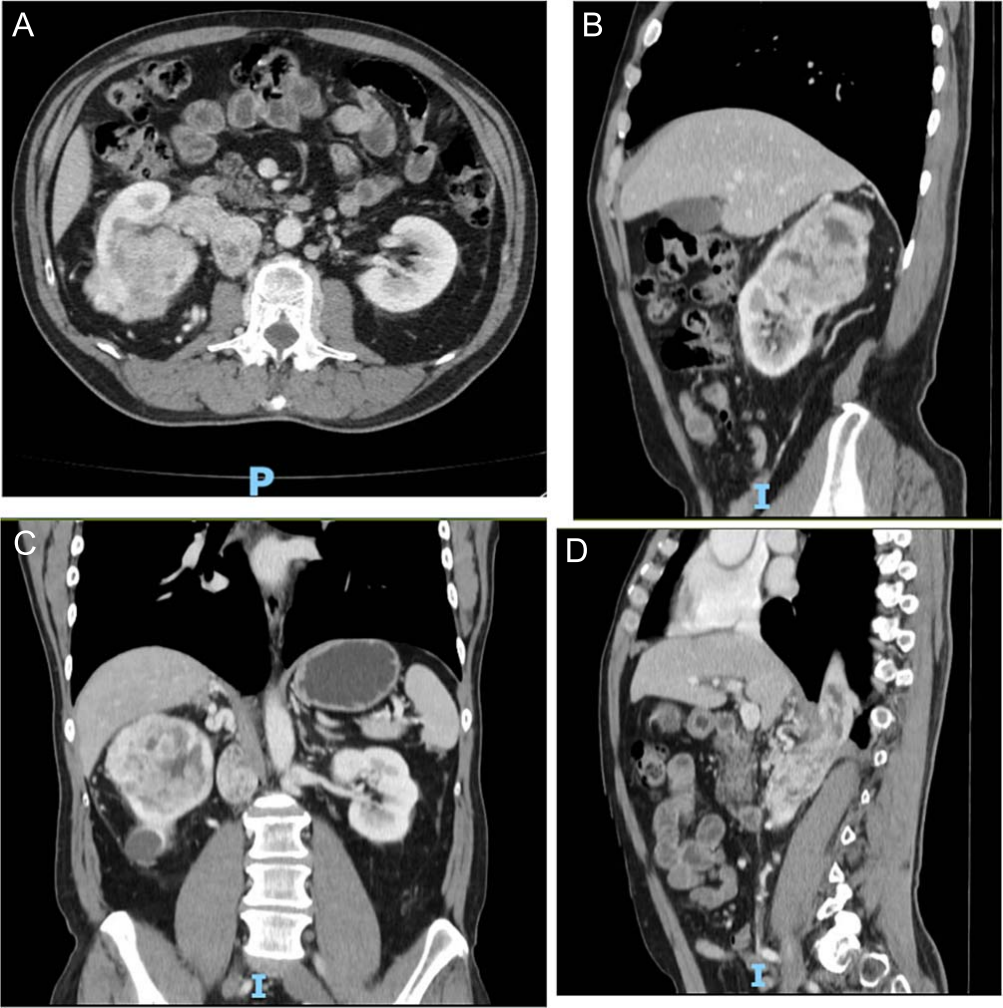
(A–D) Abdominopelvic contrast-enhanced computed tomography prior to neoadjuvant therapy showing a lesion in the right kidney with associated mural thrombus extends through the azygos vein in the sagittal section.

The authors present the case of a 60-year-old male patient with a recent diagnosis of T2a melanoma on the back, with no indication for adjuvant therapy, who underwent an abdominopelvic computed tomography with an incidental finding of a hypervascular solid mass in the right lower renal pole, compatible with an organic lesion measuring 8.5 × 7 × 8 cm invading the renal vein and the azygos vein, with intrathoracic extension (Fig 2A–D). The patient presents a vascular anomaly characterized by agenesis of the retrohepatic portion of the IVC. The hepatic veins drain directly into the right atrium, while the azygos vein serves as an alternative venous pathway, draining into the superior vena cava. A thrombus extends through the azygos vein into its intrathoracic portion.

A biopsy of the renal lesion confirmed clear cell carcinoma.

In a multidisciplinary approach, it was decided to initiate 3 months of neoadjuvant therapy with Nivolumab and Cabozantinib.

**Figure 3:**
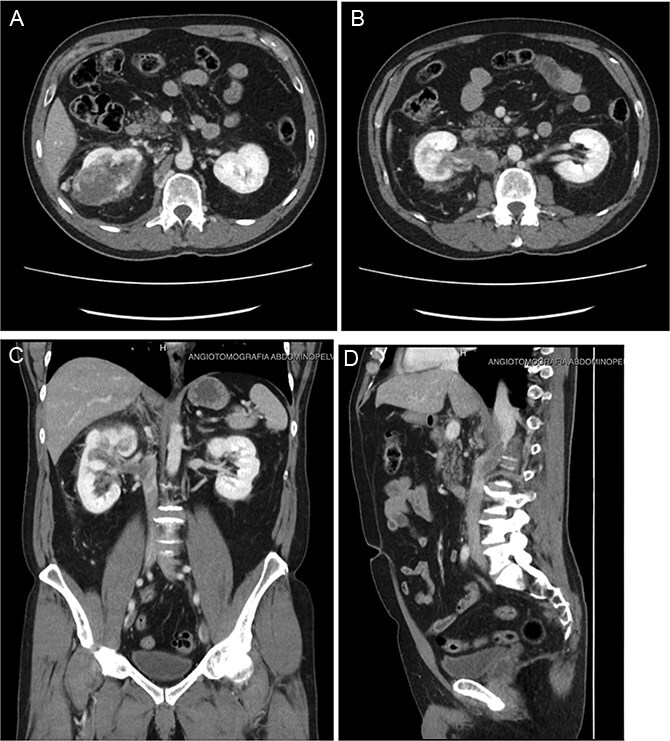
(A–D) Abdominopelvic contrast-enhanced computed tomography post-neoadjuvant therapy showed a significant reduction in tumor size and the involvement of the thrombus in the azygos vein.

Follow-up CT imaging (Fig. 3A–D) showed a reduction in both the tumor and thrombus size, leading to the classification of the thrombus from Stage IV to Stage III according to the Mayo Clinic classification.

Based on these post-treatment findings, a joint decision with Urology was made to avoid a thoraco-phreno-laparotomy, opting instead for an extended supra-infraumbilical right subcostal laparotomy.

### Surgical procedure

The extended supra-infraumbilical right subcostal laparotomy was performed, beginning with hepatic mobilization until the azygos vein was identified. Agenesis of the renal and hepatic IVC was noted, with the azygos vein replacing the cava. Once mobilized, the azygos vein was repaired along with the suprahepatic veins, which drain directly into the right atrium, and the hepatic pedicle.

The right colon-epiploic dissection was performed, revealing the right kidney with a tumor. A right radical nephrectomy was carried out by the urology team.

Next, azygotomy was performed to extract the thrombus by clamping the cava at the infrarenal level, excluding the left renal vein.

After thrombus removal, the azygos vein was reconstructed with a falciform ligament patch (Fig. 5).

### Postoperative course

The patient had a favorable postoperative recovery in the intensive care unit, progressing to a diet as tolerated, and was discharged on the 7th day anticoagulated with Enoxaparin 60 mg every 12 hours.

Ten days later, the patient was readmitted due to bleeding at the surgical site evidenced on a computed tomography scan showing a retroperitoneal hematoma, which was managed with medical treatment and anticoagulation adjustment without further complications.

**Figure 4:**
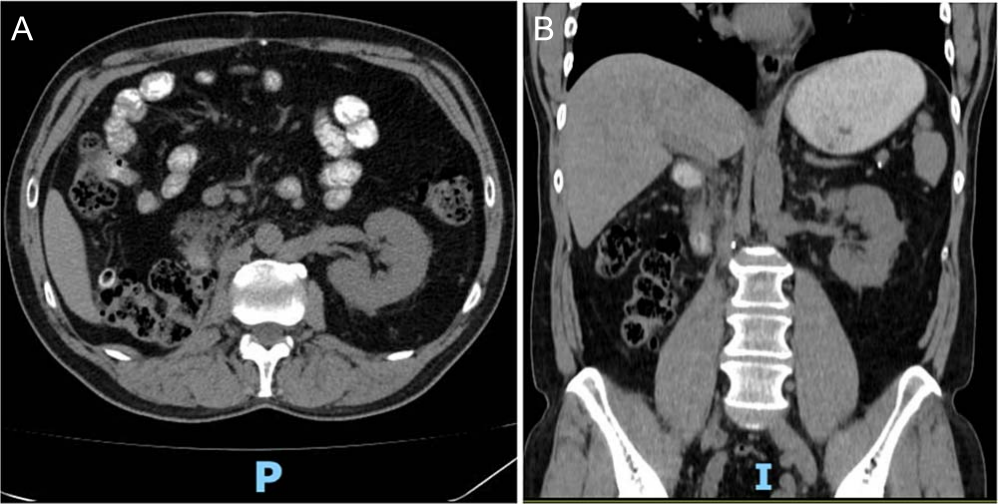
(A, B) Postoperative abdominopelvic tomography (6 months later) showing post-surgical changes consistent with right radical nephrectomy and resolution of the hematoma.

Postoperative abdominopelvic tomography (6 months later) showed post-surgical changes consistent with right radical nephrectomy and resolution of the hematoma with no evidence of recurrence (Fig. 4A–B).

**Figure 5:**
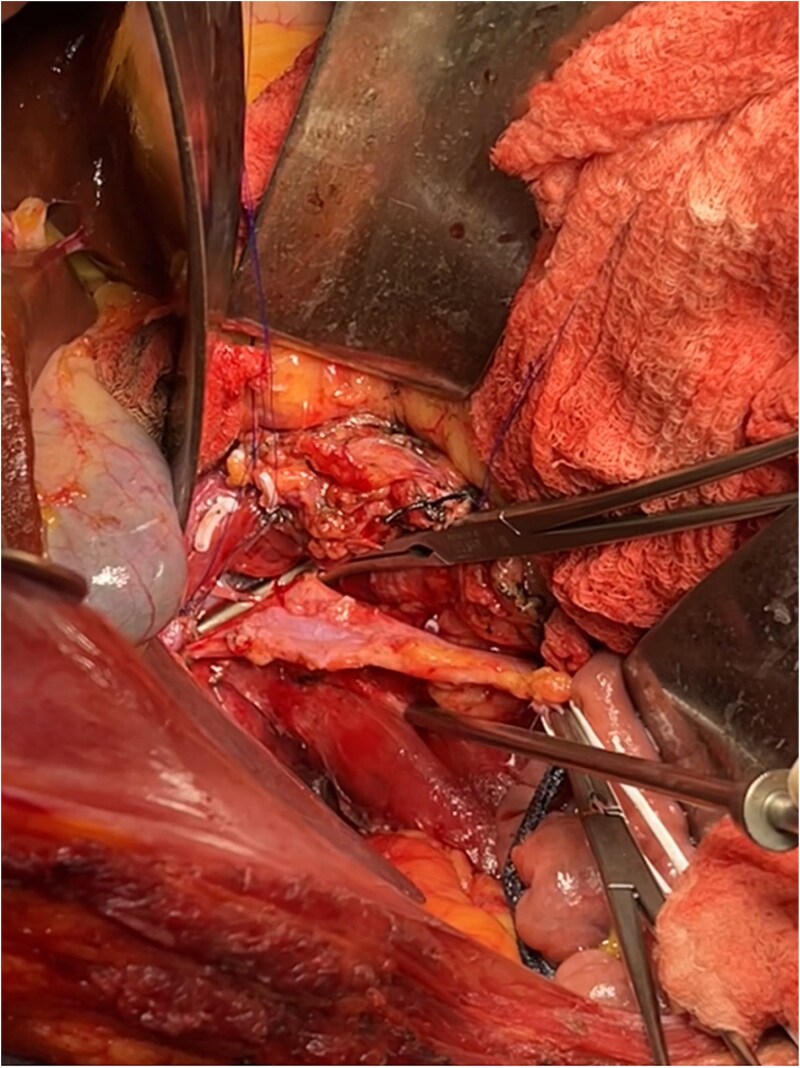
Intraoperative image following the resection of the renal tumor with thrombectomy of the azygos vein and exposure of the vena cava.

## Discussion

As previously mentioned, kidney cancer accounts for ~3% of cancer-related deaths worldwide. In Argentina, it is the fifth most common cancer, following breast, prostate, colorectal, and lung cancers. Clear cell renal carcinoma (ccRCC) represents about 90% of malignant renal tumors.

Established risk factors for ccRCC include increasing age—most commonly diagnosed between the ages of 60 and 70—smoking, and obesity. Additionally, having a first-degree relative with ccRCC is associated with an increased risk, while physical activity and moderate alcohol consumption have been identified as potential protective factors.

Rojas *et al* [6] analyzed 32 patients with stage T3b and T3c renal cancer associated with IVC TT, treated between 2001 and 2021. According to the Mayo Clinic classification, tumor thrombi are categorized as follows:

* Type I: Thrombus confined to the renal vein or extending <2 cm into the IVC.

* Type II: Thrombus extends >2 cm into the IVC but below the hepatic veins.

* Type III: Thrombus reaches the retrohepatic IVC.

* Type IV: Thrombus extends above the diaphragm or into the right atrium..

In their series, 9% had Type I thrombus, 31% Type II, 25% Type III, and 16% Type IV. Notably, this reflects a relatively high frequency of retrohepatic and supradiaphragmatic involvement. The reported intraoperative mortality was low (3%), with 19% of patients experiencing Clavien–Dindo grade III complications and 9% requiring reoperation. Overall survival was 10 months, with a 2-year cancer-specific survival rate of 40%.

Regarding neoadjuvant therapy, particularly the combination of immune checkpoint inhibitors and tyrosine kinase inhibitors, Hara *et al*. [7] reported improved disease-free survival and overall survival in patients receiving this treatment. Moreover, 52.6% of patients demonstrated a decrease in thrombus level according to the Mayo classification, along with a 60% reduction in tumor size, decreased intraoperative blood loss, and shorter operative times. [8–10]

We present the case of a patient with a rare vascular malformation—agenesis of the IVC with continuation into the azygos vein—subsequently invaded by a renal TT. Azygos vein invasion in this context is exceptionally rare, with limited reports in the literature, mostly consisting of isolated case reports.

Given the absence of specific classification systems or management guidelines for this anatomical variant, a multidisciplinary approach was essential. Neoadjuvant therapy was selected as the most appropriate initial strategy to reduce both tumor size and thrombus extension, thereby facilitating a curative-intent surgical resection and improving oncologic outcomes. The procedure was successful, with only minor complications, and follow-up imaging at 6 months postoperatively showed no evidence of disease recurrence.

## Conclusion

Multidisciplinary management is crucial in this type of pathology, as preoperative treatment with neoadjuvant therapy and proper surgical approach by experienced surgeons has been shown to reduce morbidity and mortality in such cases.

This case report highlights the importance of complementary surgical treatment alongside systemic therapy to achieve curative outcomes.

## References

[1] Jurado A, Romeo A, Gueglio G, et al. Current trends in management of renal cell carcinoma with venous thrombus extension. Curr Urol Rep 2021;22:23. 10.1007/s11934-021-01036-y 33554309

[2] Tabbara MM, González J, Martucci M, et al. Current approaches in surgical and immunotherapy-based management of renal cell carcinoma with tumor thrombus. Biomedicines 2023;11:204. 10.3390/biomedicines11010204 PMC9855836 36672712

[3] Field CA, Cotta BH, Jimenez J, et al. Neoadjuvant sunitinib decreases inferior vena caval thrombus size and is associated with improved oncologic outcomes. A multicenter comparative analysis. Clin Genitourin Cancer 2019;17:e505–12. 10.1016/j.clgc.2019.01.013 30808547

[4] Vucicevic Z, Degoricija V, Alfirevic Z, et al. Inferior vena cava agenesia and a massive bilateral iliofemoral venous thrombosis. Angiology 2008;59:510–3. 10.1177/0003319707305350 18388095

[5] Quero-Valenzuela F, Piedra-Fernández I, Hernández-Escobar F. Azygos lobe and inferior vena cava agenesis: a rare association of two uncommon malformations. Arch Bronconeumol (Engl Ed) 2020;56:114. 10.1016/j.arbres.2019.02.021 30967280

[6] Rojas PA, Bravo JC, Navarro R, et al. Nefrectomía radical con trombectomía de vena cava: 20 años de cirugías por tumor renal. Rev Med Chil 2022;150:994–9. 10.4067/S0034-98872022000800994 37358146

[7] Hara T, Suzuki K, Okamura Y, et al. Impact of neoadjuvant therapy on prognosis in renal cell carcinoma with inferior vena cava thrombus. Urol Oncol 2025;43:178–85. 10.1016/j.urolonc.2024.10.017 39537443

[8] Strugnell M, Gibson M, Hopkins R, et al. Renal cell carcinoma vertebral body metastasis extending into the azygos venous system causing superior vena cava obstruction. Br J Radiol 2005;78:65–7. 10.1259/bjr/68967544 15673535

[9] Probst S, Seltzer A, Chachoua A, et al. Azygos venous tumor thrombus from renal cell carcinoma detected by F-18 FDG PET/CT. Clin Nucl Med 2010;35:832–3. 10.1097/RLU.0b013e3181ef0bae 20838303

[10] Aurangabadkar H, Ali Z. Unusual metastatic sites from renal cell carcinoma detected by 18F-FDG PET/CT scan. Clin Nucl Med 2013;38:e471–3. 10.1097/RLU.0b013e31828680a6 23603603

[11] Bass JE, Redwine MD, Kramer LA, et al. Spectrum of congenital anomalies of the inferior vena cava: cross-sectional imaging findings. Radiographics 2000;20:639–52. 10.1148/radiographics.20.3.g00ma09639 10835118

